# Effects of IFN‐γ on immune cell kinetics during the resolution of acute lung injury

**DOI:** 10.14814/phy2.14368

**Published:** 2020-02-15

**Authors:** Jason R. Mock, Miriya K. Tune, Catherine F. Dial, Jose Torres‐Castillo, Robert S. Hagan, Claire M. Doerschuk

**Affiliations:** ^1^ Division of Pulmonary Diseases and Critical Care Medicine Department of Medicine University of North Carolina Chapel Hill North Carolina; ^2^ Marsico Lung Institute University of North Carolina Chapel Hill North Carolina; ^3^ Center for Airways Disease University of North Carolina Chapel Hill North Carolina

**Keywords:** acute lung injury, immune cell kinetics, interferon‐gamma, regulatory T cells, resolution

## Abstract

The immunologic responses that occur early in the acute respiratory distress syndrome (ARDS) elicit immune‐mediated damage. The mechanisms underlying the resolution of ARDS, particularly the role of signaling molecules in regulating immune cell kinetics, remain important questions. Th1‐mediated responses can contribute to the pathogenesis of acute lung injury (ALI). Interferon‐gamma (IFN‐γ) orchestrates early inflammatory events, enhancing immune‐mediated damage. The current study investigated IFN‐γ during resolution in several experimental models of ALI. The absence of IFN‐γ resulted in altered kinetics of lymphocyte and macrophage responses, suggesting that IFN‐γ present in this microenvironment is influential in ALI resolution. Genetic deficiency of IFN‐γ or administering neutralizing IFN‐γ antibodies accelerated the pace of resolution. Neutralizing IFN‐γ decreased the numbers of interstitial and inflammatory macrophages and increased alveolar macrophage numbers during resolution. Our results underline the complexity of lung injury resolution and provide insight into the effects through which altered IFN‐γ concentrations affect immune cell kinetics and the rate of resolution. These findings suggest that therapies that spatially or temporally control IFN‐γ signaling may promote ALI resolution. Identifying and elucidating the mechanisms critical to ALI resolution will allow the development of therapeutic approaches to minimize collateral tissue damage without adversely altering the response to injury.

## Graphical abstract

Neutralizing IFN‐γ decreased the numbers of interstitial and inflammatory macrophages and increased the alveolar macrophage numbers during resolution. These results underline the complexity of lung injury resolution and provide insight into the effects through which altered IFN‐γ concentrations affect immune cell kinetics and the rate of resolution. These findings suggest that therapies that spatially or temporally control IFN‐γ signaling may promote ALI resolution.

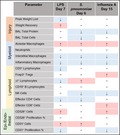

## 1 INTRODUCTION

Events such as pneumonia, inhalational injury, trauma, or sepsis can damage the lung, which consequently impedes its primary function of gas exchange. These events can result in acute respiratory distress syndrome (ARDS), a clinical syndrome that has significant morbidity and mortality in the United States each year (Phua et al., 2009; Thompson, Chambers, & Liu, 2017). The most common risk factors for ARDS are pneumonia or sepsis (Tsushima et al., 2009).

The mechanisms underlying the pathogenesis of ARDS have been significantly advanced in recent years, and the immunologic responses that occur early in ARDS elicit immune‐mediated alveolar damage (Huang et al., 2005; Ramana et al., 2015; Theron, Huang, Chen, Liu, & Lei, 2005; Wlodarczyk, Kraft, Chen, Kenney, & Selin, 2013). Th1‐mediated responses can contribute to the pathogenesis of acute lung injury (ALI) (Aggarwal, King, & D'Alessio, 2014). A critical Th1 mediator, interferon‐gamma (IFN‐γ), enhances inflammatory and immune‐mediated damage (Clark et al., 1998; Rijneveld et al., 2002; Schultz, Rijneveld, Speelman, Deventer, & Poll, 2001; Theron et al., 2005).

After an acute injury, repair and the resolution of the damage must occur for adequate gas exchange to return. Resolution from injury is not merely relief from injurious stimuli, but rather an actively regulated program involving removal of apoptotic neutrophils, remodeling of the matrix, reabsorption of protein‐rich alveolar fluid, and repair of the endothelial and epithelial cells of the alveolar space (Huynh, Fadok, & Henson, 2002; Kieran, Maderna, & Godson, 2004; Savill, 1997; Serhan & Savill, 2005). Less is known about what effects immunologic signaling molecules, such as IFN‐γ, have on immune cell kinetics during resolution of ALI.

IFN‐γ is a type II IFN and the sole member of this family. A broad range of cells produce IFN‐γ, and significant contributing populations include CD4^+^ T helper type I lymphocytes, CD8^+^ cytotoxic lymphocytes, group 1 innate lymphoid cells (ILC1s) and NK cells, as well as antigen‐presenting cells, B lymphocytes, neutrophils, and NK T lymphocytes (Fenimore, 2016; Riggan, Freud, & O'Sullivan, 2019; Varma, Lin, Toliver‐Kinsky, & Sherwood, 2002). IFN‐γ production by neutrophils occurs during *Streptococcus pneumoniae* pneumonia (Gomez et al., 2015). Cytokines such as IL‐12 and IL‐18 are upstream signals for IFN‐γ production, whereas negative regulators of IFN‐γ expression include glucocorticoids, IL‐4, IL‐10, and TGFβ (Fenimore, 2016). IFN‐γ is vital for host immunity against intracellular pathogens, whereas its role in host defense toward extracellular pathogens is more variable (Moldoveanu et al., 2009).

The receptor for IFN‐γ is comprised of two IFNGR1 chains and two IFNGR2 chains. IFNGR1 is expressed on most cells at moderate levels, while IFNGR2 is expressed at lower levels; however, IFNGR2 expression can be regulated in specific cell types (Bach, Aguet, & Schreiber, 1997; Bernabei et al., 2001; Fenimore, 2016; Green, Young, & Valencia, 2017; Londino et al., 2017). *Ifng* ^−/−^ and *Ifngr1* ^−/−^ mice develop normally and have a typical immune system, but both genotypes have defects in resistance to some infectious agents, including mycobacterial species, some viruses, and bacteria; conversely, the *Ifngr1* ^−/−^ mice are more resistant to LPS (Car et al., 1994; Nakamura et al., 2000; Schroder, Hertzog, Ravasi, & Hume, 2004).

In this study, we sought to determine the changes in the development and resolution of ALI that result from the deficiency of IFN‐γ. Our studies focused on LPS‐induced ALI, examining both day 4 and day 7. Day 4 post‐injury is the time of peak inflammation when the numbers of neutrophils and inflammatory macrophages are peaking. By day 7 post‐injury, resolution is underway; type II epithelial cell (AT2) proliferation is high, lymphocyte numbers are increasing, and neutrophils and inflammatory macrophages are decreased. Two other stimuli that are important common causes of lung injury (*S. pneumoniae* and H1N1 influenza) were also studied to identify and compare the effects of IFN‐γ deficiency at a time point during resolution when mice have regained much of their weight loss (Arpaia et al., 2015; Gomez et al., 2017; Matute‐Bello, Frevert, & Martin, 2008). These studies identified the contribution of IFN‐γ both to lung injury and to changes in immune cell kinetics during resolution from lung injury.

## 2 METHODS

### 2.1 Mice

C57BL/6 wild‐type (WT) and *Ifng* ^−/−^ mice were obtained from Jackson Laboratory (Dalton et al., 1993). Mice were housed in ventilated cages, and colonies were maintained within a pathogen‐free facility at the University of North Carolina at Chapel Hill. Animal procedures and protocols were approved by the University of North Carolina Animal Care and Use Committee.

### 2.2 Preparation of Mice for LPS administration

Eight to twelve‐week‐old male and female mice were anesthetized and administered lipopolysaccharide, *Escherichia coli* LPS O55:B5 (3 mg/kg) (Sigma‐Aldrich), as previously described (D'Alessio et al., 2009; Dial, Tune, Doerschuk, & Mock, 2017; Mock et al., 2014).

### 2.3 Bacterial pneumonia

*Streptococcus pneumoniae* (*S. pneumoniae*; *serotype* 19, ATCC 49619) was purchased from American Type Culture Collection. Bacteria were grown overnight at 37°C in 5% CO_2_ on blood agar plates, 5% sheep blood in tryptic soy agar (ThermoFisher). 10–20 colonies were then suspended in Todd‐Hewitt Broth (Becton Dickinson) supplemented with 17% (v/v) Fetal Bovine Serum (ThermoFisher) and incubated at 37°C with shaking at 225 rpm for several hours until an OD_600_ 0.3 was reached as previously described (D'Alessio, 2018). The media was distributed into 1 ml aliquots and flash‐frozen in liquid nitrogen before storage at −80°C (D'Alessio, 2018). Pneumonia was induced by intratracheal instillation of the thawed bacterial suspension at a dose of 2 µl/g mouse body weight. Colony‐forming units (CFU) in bacterial suspensions were subsequently determined by plating serial dilutions of the bacterial suspension on blood agar plates. The range of CFUs was 4.79–7.54 × 10**^6^** CFU/mouse.

### 2.4 Influenza infection

Influenza A/PR/8/34 H1N1 (PR8) was purchased from Charles River (Norwich, CT; Catalog # 10100374). The viral administration has been dose‐optimized for eliciting a robust inflammatory response and modest mortality of 10 to 15 percent, facilitating a better study of the resolution phase of ALI (Kanegai et al., 2016; Mock et al., 2014). The virus was suspended and diluted in PBS and stored at −80°C at 2 × 10^8^ egg‐infective dose/ml (EID). Pneumonia was induced by intratracheal instillation of the thawed viral suspension diluted in PBS to 5 × 10^5^ EID/ml. Mice received 40 μl of this dilution intratracheally.

### 2.5 RNA isolation and analysis of Influenza A *M2* gene expression

At time points after influenza A infections, lungs were snap‐frozen in liquid nitrogen and RNA obtained to quantitate viral expression as previously described (Hagan, Torres‐Castillo, & Doerschuk, 2019).

### 2.6 In vivo antibody‐mediated neutralization of IFN‐ γ

WT animals were given 20 µg/dose/mouse of intraperitoneal injections of a rat monoclonal anti‐ IFN‐γ antibody (Clone AN‐18) or isotype control (IgG1 κ isotype, BioLegend) on days 1, 2, and 3 post LPS administration. See Table S1 for clone and catalog numbers.

### 2.7 Analysis of BAL fluid

BAL was performed by cannulating the trachea with a 20‐gauge catheter and lavaging the lungs with PBS as previously described (D'Alessio et al., 2009; Mock et al., 2014).

### 2.8 Lung morphology

Lungs were inflated to 25 cm H_2_O and fixed with 10% formalin (ThermoFisher) for histological evaluation by hematoxylin and eosin staining (D'Alessio et al., 2009; Mock et al., 2019).

### 2.9 Preparation of lung single‐cell suspensions for flow cytometry analysis

At endpoints, uninjured, baseline (control), 4, 6, 7, or 15 days after instillation, mice were euthanized by isoflurane overdose as previously described (Dial et al., 2017). Lungs were removed, and single‐cell suspensions were prepared for flow cytometry analysis as described previously (Dial et al., 2017; Mock et al., 2019). See Table S1 for clone and catalog numbers. The neutrophils and macrophage subpopulations were identified through gating, as demonstrated in Figure S2 and adapted from (Misharin, Morales‐Nebreda, Mutlu, Budinger, & Perlman, 2013), and these four populations were immunophenotyped as:

1. Neutrophils: CD45^+^CD64^+^CD24^+^CD11b^+^SiglecF^‐^Ly6G^+^
2. Alveolar macrophages: CD45^+^CD64^+^CD11c^+^MHCII^±^SiglecF^+^Ly6C^‐^
3. Interstitial macrophages: CD45^+^CD64^+^CD11b^+^MHCII^+^SiglecF^‐^Ly6G^‐^Ly6C^‐^
4. Inflammatory macrophage: CD45^+^CD64^+^CD11b^+^MHCII^+^SiglecF^‐^Ly6G^‐^Ly6C^+^.

### 2.10 Statistics

Analysis of data utilized comparisons using the Mann–Whitney rank‐sum test or one‐way or two‐way ANOVA with Tukey's or Holm–Sidak methods for multiple *t*‐test comparisons. Data are expressed as the mean ± standard error of the mean. Statistical analysis was performed using GraphPad Prism 7 software. Statistical difference was accepted at *p* < .05.

## 3 RESULTS

### 3.1 The effects of IFN‐γ on immune cell kinetics during ALI induced by LPS

**Figure 1:**
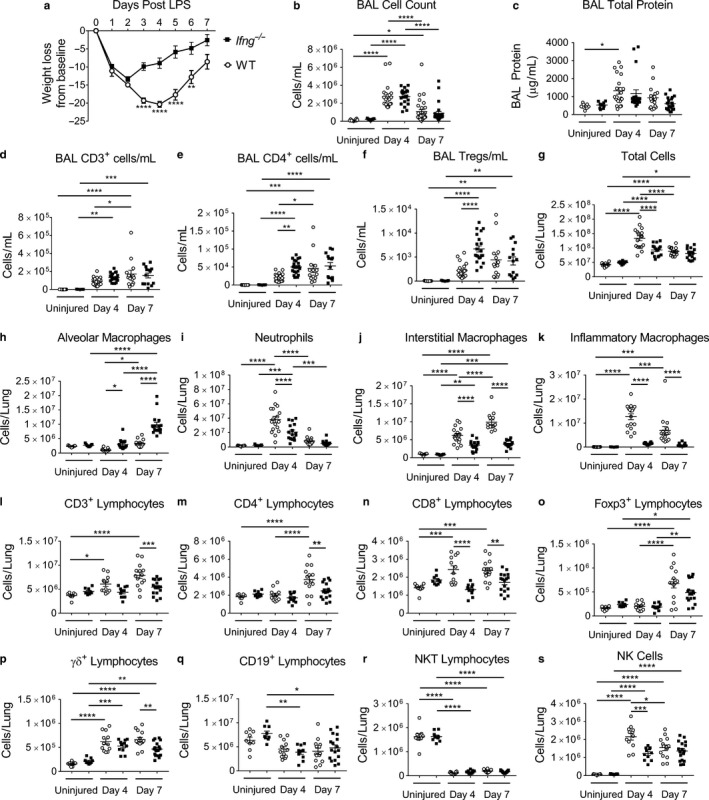
Interferon‐γ null mice have altered ALI immune cell populations and resolution kinetic parameters. *Ifng* ^−/−^ or WT mice were challenged with intratracheal LPS administered on day 0. Uninjured mice or mice after injury were examined at day 4 or 7 post LPS for injury parameters or immunophenotyping in the BAL compartment or enzymatic single‐cell lung suspensions. (a) Body weight relative to baseline determined after injury (*n* = 38–49 per group for *Ifng* ^−/−^ mice; *n* = 35 – 49 per group for WT mice, combining four independent experiments). (b) BAL cell counts and (c) BAL total protein determined for uninjured mice and also on day 4 or 7 after LPS‐induced injury between *Ifng* ^−/−^ or WT mice (*n* = 9–31 per genotype, combined from 2 or more independent experiments). (d–f) BAL immunophenotyping of CD3^+^, CD4^+^, and Tregs in uninjured mice and also at days 4 or 7 post LPS administration (*n* = 9–22 per group, combined from 2 or more independent experiments). (g) Total lung cell count obtained from lung digests from uninjured mice or obtained from mice at day 4 or 7 post LPS treatment (*n* = 9–17 per genotype per group, combined data of two independent experiments). (h–k) Changes in macrophage subpopulations and neutrophils as total numbers in single‐cell suspensions determined by flow cytometry with gating adapted from Misharin et al. (2013). (l–s) Changes in CD3^+^, CD4^+^, CD8^+^, Foxp3^+^, γδ^+^, CD19^+^, NK T lymphocyte subsets and NK cells as total numbers in single‐cell suspensions determined using a previously published lymphocyte flow cytometric panel and gating approach (Mock et al., 2019) (*n* = 9–17 per group, combined from two independent experiments). Data are expressed as the mean ± *SEM*. *p* values determined by two‐way ANOVA with the Sidak's multiple comparison test. *p < .05, **p < .01 ***p < .001, ****p < .0001

In naïve (uninjured) mice, there were no differences in the numbers of any immune cell types examined between *Ifng* ^−/−^ and WT mice in either the BAL or the lung digest (Figure 1 and Figure S1). Mice lacking IFN‐γ during LPS‐induced ALI had significantly less weight loss compared to control mice (Figure 1a). No difference in mortality occurred between genotypes (Figure S1a). Lung histopathology at day 4 demonstrated less cellularity in the *Ifng* ^−/−^ mice compared to WT (Figure S1b). The BAL cell count and total protein at time points of peak injury (Day 4) and during resolving lung injury (Day 7) were similar between genotypes (Figure 1b and c). Interestingly, in the BAL compartment at day 4 post LPS, a time point of peak inflammation, *Ifng* ^−/−^ mice contained more CD4^+^ and Treg cells than WT mice; however, by day 7, the numbers of these lymphocytes were similar between genotypes (Figure 1d–f).

The lungs of *Ifng* ^−/−^ mice had less cellularity than WT mice, as demonstrated by the total number of cells obtained from lung digests at 4 days post LPS; however, no differences were present between genotypes by day 7 (Figure 1g). In these lung digests, *Ifng* ^−/−^ mice had more alveolar macrophages on Day 4 and 7 post LPS (Figure 1h, please see Methods section for a complete definition of cell populations). *Ifng* ^−/−^ mice had fewer neutrophils at day 4, and fewer interstitial and inflammatory macrophages on both days 4 and 7 post LPS (Figure 1i–k); representative gating is shown in Figure S2 (adapted from (Misharin et al., 2013)). In the BAL compartment, *Ifng* ^−/−^ mice also had more alveolar macrophages on Day 7 post LPS and fewer neutrophils and interstitial and inflammatory macrophages (data not shown). Interestingly, the increase in inflammatory macrophages, which are recruited from the bone marrow, was entirely prevented by the absence of IFN‐γ.

In the same lung digests, *Ifng* ^−/−^ lungs contained fewer CD8^+^ lymphocytes at day 4 and 7 post LPS, and fewer CD3^+^ and CD4^+^ lymphocytes on day 7 (Figure 1l–n), as identified by flow cytometry with gating as previously reported (Mock et al., 2019). There was no significant difference in the number of lung Foxp3^+^ Tregs between genotypes, and Tregs increased in both genotypes on day 7 during resolution (Figure 1o). The number of γδ^+^ lymphocytes was lower at day 7 post LPS in *Ifng* ^−/−^ mice (Figure 1p). No significant difference was detected in CD19^+^ lymphocytes or NK T lymphocytes on either day 4 or 7 post‐injury between genotypes (Figure 1q and r). The number of NK cells was lower on day 4 (Figure 1s). The increases in CD4^+^ and CD8^+^ lymphocytes were entirely prevented by the absence of IFN‐γ, while the increases in γδ^+^ lymphocytes and NK cells appear only partially due to IFN‐γ. In BAL fluid at day 7, only γδ^+^ lymphocytes and CD19^+^ cells were significantly different between genotypes and present in higher numbers in *Ifng* ^−/−^ mice (data not shown).

The percentage of CD4^+^ lymphocytes that were activated or effector CD4^+^ cells, as determined by CD44^+^ CD62L^lo^ surface marker expression, was lower only at day 7 in *Ifng* ^−/−^ mice when compared to WT (Figure S1c–f). The percentage of effector CD8^+^ lymphocytes was similar in both genotypes at all time points examined. The percentage of lung CD4^+^ lymphocytes that co‐expressed Foxp3 (percentage of total CD4^+^ cells that were Foxp3^+^) did not differ between genotypes but did increase at 7 days post LPS (Figure S1g).

Interestingly, when the numbers and percentages of proliferating endothelial (CD31^+^) and epithelial (CD326^+^) cells were examined, *Ifng* ^−/−^ mice had increased total numbers of both CD31^+^ and CD326^+^ cells at day 7 post‐injury compared to WT mice (Figure S1h and k). The percentage and number of proliferating epithelial cells did not differ between genotypes; however, on day 4, the percentage and number of proliferating endothelial cells were higher in *Ifng* ^−/−^ mice compared to WT (Figure S1h–m). Curiously, both the percentage and the number of proliferating endothelial cells were higher in WT mice by Day 7, but this increase in proliferation and number occurred earlier in *Ifng* ^−/−^ mice by day 4 (Figure S1l and m). Taken together, this suggests that IFN‐γ has different effects on individual cell types.

### 3.2 The effects of IFN‐γ on immune cell kinetics during ALI induced by *S. pneumoniae*

**Figure 2:**
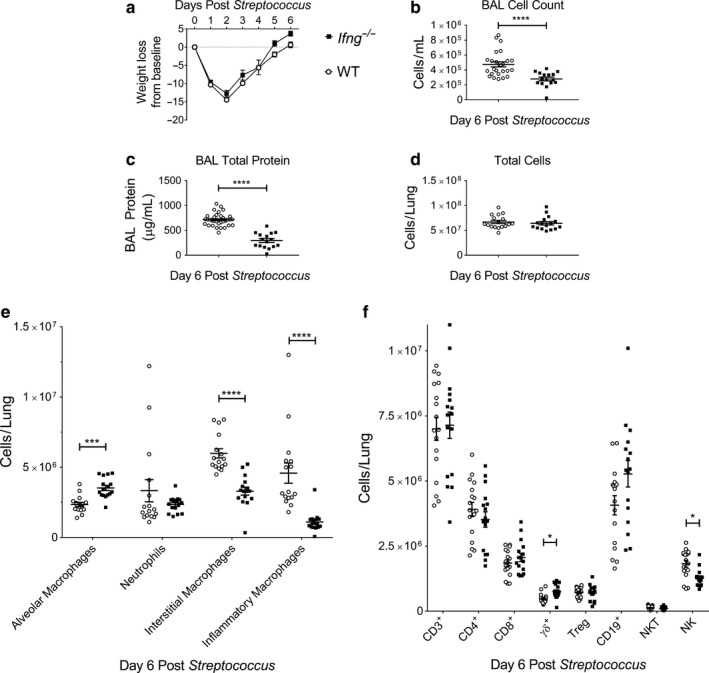
Interferon‐γ null mice have altered immune cell populations and resolution kinetic parameters after *Streptococcus* *pneumoniae* ALI. *Ifng* ^−/−^ or WT mice were challenged with intratracheal *Streptococcus pneumoniae* (*Sp*) administered on day 0. Mice were examined at day 6 post‐*Sp* for injury parameters or immunophenotyping in lung digests. (a) Body weight relative to baseline determined after injury (*n* = 21 for *Ifng* ^−/−^ mice; *n* = 19 for WT mice, combining two independent experiments). (b) BAL cell counts and (c) BAL total protein determined on day 6 after *Sp*‐induced injury between *Ifng* ^−/−^ or WT mice (*n* = 16–33 per genotype, combined from two or more independent experiments). (d) Total lung cell count obtained from lung digests obtained at day 6 post‐*Sp* treatment exhibit similar cellularity (*n* = 16–17 per genotype, combined data of two independent experiments). (e) Changes in lung macrophage subpopulations and neutrophils as total numbers in single‐cell suspensions determined by flow cytometry with gating adapted from Misharin et al. (2013). (f) Changes in CD3^+^, CD4^+^, CD8^+^, Foxp3^+^, γδ^+^, CD19^+^, NK T lymphocyte subsets and NK cells as total numbers in single‐cell suspensions determined using a previously published lymphocyte flow cytometric panel and gating approach (Mock et al., 2019). Data are expressed as the mean ± *SEM*. *p* values determined by either Mann–Whitney rank sum test or one‐way ANOVA with the Holm–Sidak, multiple comparison tests. *p < .05, ***p < .001, ****p < .0001

Mice given ALI induced by *Streptococcus pneumoniae* (*Sp*) were studied 6 days after infection, a time point during resolution when infected mice have regained much of their weight loss (Gomez et al., 2017; Matute‐Bello et al., 2008) Mice lacking IFN‐γ had a similar weight loss and return to baseline weight by 6 days post‐*Sp*, compared to WT mice (Figure 2a). In prior reports, similar inocula of *Sp* did not demonstrate a difference in bacterial clearance between genotypes (Yamada et al., 2011). There was no difference in survival between genotypes (Figure S3a). Lung histopathology at day 6 appeared similar between genotypes (Figure S3b). *Ifng* ^−/−^ mice had a lower total BAL cell count and total protein concentration than WT mice (Figure 2b and c). When comparing myeloid and lymphoid cell numbers in the BAL compartment, only inflammatory macrophages were significantly different between genotypes and present in lower numbers in *Ifng* ^−/−^ mice (data not shown).

The lungs of *Ifng* ^−/−^ mice had similar cellularity, as demonstrated by the total number of cells obtained from lung digests at 6 days post‐*Sp* (Figure 2d). In lung digests, *Ifng* ^−/−^ mice had fewer lung interstitial and inflammatory macrophages and more alveolar macrophages than WT mice (Figure 2e, Figure S2). In the same lung digests, *Ifng* ^−/−^ lungs contained fewer NK cells at day 6 and more γδ^+^ lymphocytes (Figure 2f). There was no significant difference in the number of lung Foxp3^+^ Tregs between genotypes (Figure 2f).

The percentage of effector CD4^+^ lymphocytes, as determined by CD44^+^ CD62L^lo^ surface markers, was lower at day 6 in *Ifng* ^−/−^ mice (Figure S3c). The percentage of effector CD8^+^ cells was similar in both genotypes. The percentage of lung CD4^+^ lymphocytes that co‐expressed Foxp3 did not differ between genotypes (Figure S3d).

Similar to the LPS‐induced lung injury, the number of endothelial cells (CD31^+^) was higher in *Ifng* ^−/−^ mice compared to WT mice at day 6 post‐*Sp*; however, the number of epithelial cells (CD326^+^) and percent of proliferating endothelial (CD31^+^) or epithelial (CD326^+^) cells were similar between genotypes (Figure S3e–j). Taken together, IFN‐γ similarly affects the macrophage subpopulations after *Sp* and LPS‐induced ALI, but does not contribute significantly to the lymphoid response to *Sp*.

### 3.3 The effects of IFN‐γ on immune cell kinetics during ALI induced by Influenza A

**Figure 3:**
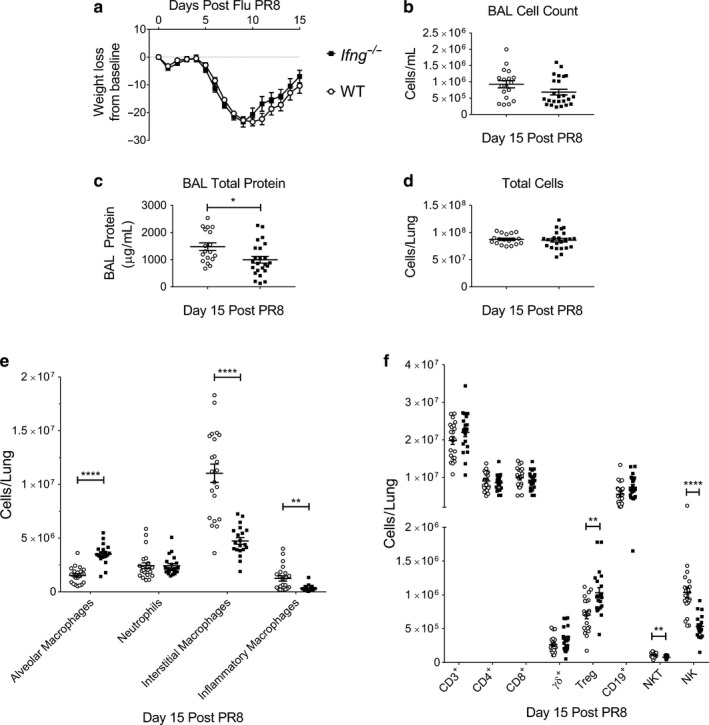
Interferon‐γ null mice have altered immune cell populations and resolution kinetic parameters in Influenza‐induced ALI. *Ifng* ^−/−^ or WT mice were challenged with intratracheal Influenza A/PR/8/34 H1N1 (PR8) administered at day 0. Mice were examined at day 15 post PR8‐induced for injury parameters or immunophenotyping in lung digests. (a) Body weight relative to baseline determined after injury (*n* = 26 for *Ifng* ^−/−^ mice; *n* = 31 for WT mice, combining three independent experiments). (b) BAL cell counts and (c) BAL total protein determined on day 15 after PR8‐induced injury between *Ifng* ^−/−^ or WT mice (*n* = 12–14 per genotype, combined from 2 or more independent experiments). (d) Total lung cell count obtained from lung digests obtained at day 15 post PR8 treatment exhibit similar cellularity (*n* = 12–14 per genotype, combined data of 2 independent experiments). (e) Changes in lung macrophage subpopulations and neutrophils as total numbers in single‐cell suspensions determined by flow cytometry with gating adapted from Misharin et al. (2013). (f) Changes in CD3^+^, CD4^+^, CD8^+^, Foxp3^+^, γδ^+^, CD19^+^, NK T lymphocyte subsets, or NK cells as total numbers in single‐cell suspensions determined using a previously published lymphocyte flow cytometric panel and gating approach (Mock et al., 2019). Data are expressed as the mean ± *SEM*. *p* values determined by either Mann–Whitney rank sum test or one‐way ANOVA with the Holm–Sidak, multiple comparison tests. *p < .05, **p < .01, ****p < .0001

Mice lacking IFN‐γ during influenza‐induced (PR8) ALI had similar weight loss and recovery compared to control mice, which nadired at day 10 post PR8 infection (Figure 3a). There was no difference in survival between genotypes (Figure S4a). The lung histopathology at day 15 demonstrated similar cellularity between genotypes (Figure S4b). The total BAL cell count was similar between genotypes, while the BAL total protein concentration was lower in *Ifng* ^−/−^ mice (Figure 3b and c, respectively). There was no difference in the clearance of PR8 from the lungs (Figure S4c).

The lungs of *Ifng* ^−/−^ mice had similar cellularity, as demonstrated by the total number of cells obtained in the lung digests at 15 days post‐PR8 administration (Figure 3d). In these digests, *Ifng* ^−/−^ mice had fewer lung interstitial and inflammatory macrophages and more alveolar macrophages at day 15 post PR8 (Figure 3e, Figure S2). There were no significant differences at this time point in the number of lung CD3^+^, CD4^+^, CD8^+^, CD19^+^, or γδ^+^ lymphocytes between genotypes (Figure 3f), although PR8 induced an increase in each of these cell types. However, the *Ifng* ^−/−^ lungs contained less NK cells and NK T lymphocytes and more Foxp3^+^ Tregs at day 15 post PR8 (Figure 3f). The percentage of CD4^+^ lymphocytes that expressed Foxp3 was also higher in *Ifng* ^−/−^ than WT mice (Figure S4d). In the BAL fluid, comparison of myeloid and lymphoid cell numbers revealed that only alveolar, interstitial, and inflammatory macrophages were significantly different, and the direction of the changes corresponded to those found in the lung digests (data not shown). Taken together, IFN‐γ similarly affects the macrophage subpopulations during resolution after PR8, *Sp*, and LPS ALI, and contributes more to the lymphoid response following PR8 than *Sp*.

The percentage of effector CD8^+^ lymphocytes was higher at day 15 in *Ifng* ^−/−^ mice (Figure S4e). The percentage of effector CD4^+^ cells was similar in both genotypes.

Similar to LPS*,* PR8 infection resulted in more CD326^+^ epithelial cells in *Ifng* ^−/−^ mice without any change in the percentage or number of proliferating epithelial cells (Figure S4f–h). The number of endothelial cells was similar between genotypes, but the rates and number of proliferating endothelial (CD31^+^) cells were less in the *Ifng* ^−/−^ mice compared to WT mice (Figure S4i–k).

### 3.4 Neutralizing IFN‐γ after initiation of ALI affects immune cell populations during resolution of ALI

**Figure 4:**
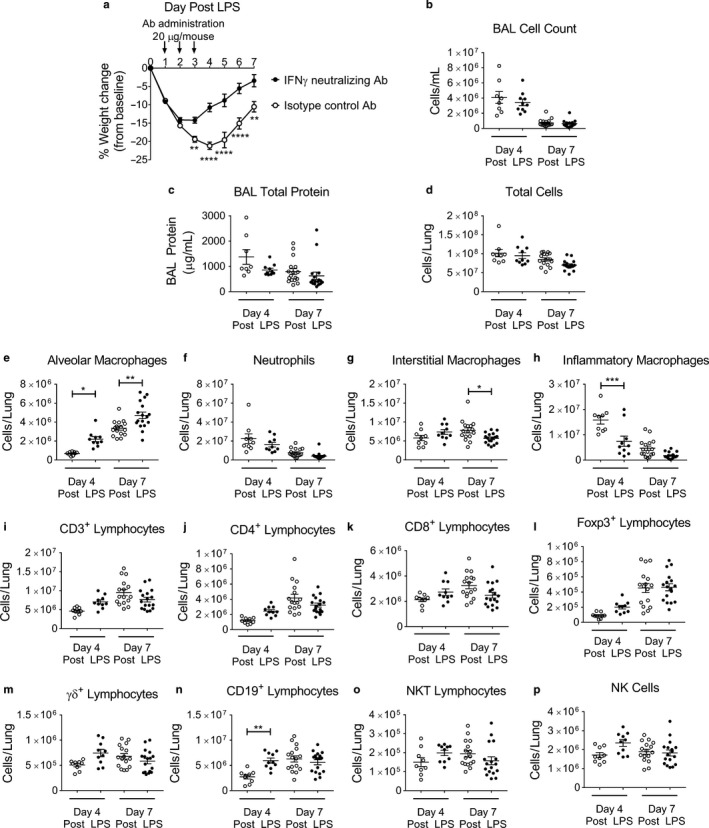
Interferon‐γ neutralization augments ALI recovery. WT mice were challenged with LPS administered at day 0 and then on days 1, 2, and 3 post LPS mice were given intraperitoneal injections of 20 μg/mouse of either an IFN‐γ neutralizing antibody or isotype control antibody. Mice were weighed daily and examined at day 4 or 7 post LPS for injury parameters or immunophenotyping in the BAL compartment or lung digests (*n* = 8–17 per group, combined from at least two independent experiments) (a) Body weight relative to baseline determined after injury. (b) BAL cell counts numbers total protein concentration, (c) BAL total protein concentration, and (d) Total lung cell counts obtained from enzymatically digested single‐cell suspension are similar between antibody administrations. (e–h) Changes in lung macrophage subpopulations and neutrophils as total numbers in single‐cell suspensions determined by flow cytometry with gating adapted from Misharin et al. (2013). (i–p) Changes in CD3^+^, CD4^+^, CD8^+^, Foxp3^+^, γδ^+^, CD19^+^, NK T lymphocytes subsets and NK cells as total numbers in single‐cell suspensions determined using a previously published lymphocyte flow cytometric panel and gating approach (Mock et al., 2019). *p* values determined by two‐way ANOVA with the Holm–Sidak multiple comparison tests. *p < .05, **p < .01 ***p < .001, ****p < .0001

To examine the hypothesis that neutralizing IFN‐γ during ALI accelerates lung resolution, we performed antibody‐mediated neutralization experiments, in which WT mice were given either an IFN‐γ neutralizing antibody or an isotype antibody control at days 1, 2, and 3 post LPS‐induced injury. Mice given the IFN‐γ neutralizing antibody had significantly less weight loss compared to isotype control mice (Figure 4a). There was no significant difference in survival between antibodies (Figure S5a). Lung histopathology at day 4 or 7 post LPS appeared similar between experimental groups (Figure S5b and c), as did total BAL cell counts and BAL total protein at days 4 and 7 (Figure 4b and c).

WT mice administered anti‐IFN‐γ antibody had similar cellularity, as demonstrated by the total number of cells in lung digests at 4 or 7 days (Figure 4d). WT mice given anti‐IFN‐γ antibody had more alveolar macrophages, similar numbers of neutrophils, fewer inflammatory macrophages at day 4, and fewer interstitial macrophages at day 7 compared to isotype controls (Figure 4e–h). No significant differences were found in the other immune cell populations, such as CD3^+^, CD4^+^ and CD8^+^ cells, Foxp3^+^ Tregs, γδ^+^ lymphocytes, NK T lymphocytes, or NK cells (Figure 4i–p).

The percentages of effector CD4^+^ and CD8^+^ lymphocytes, as determined by CD44^+^ CD62L^lo^ surface markers, were similar between the two groups (Figure S5d–f). There were also no differences in the percentage of total CD4^+^ that were Tregs at either time point (Figure S5g). Additionally, no differences in the numbers or the percent of proliferating endothelial (CD31^+^) and epithelial (CD326^+^) cells were measured between genotypes at day 7 post LPS (Figure S5h–m).

These results suggest that neutralizing IFN‐γ impacts on some immune cell populations during resolution of LPS‐induced lung injury; however, the neutralizing experiments (Figure 4) only partially mirrored the changes seen in genetically deficient mice, even at Day 4 (Figure 1, Figure S1).

## 4 DISCUSSION

This report investigates the roles of IFN‐γ in ALI induced by three stimuli by examining the effects caused by the absence of IFN‐γ during these injuries or by neutralizing IFN‐γ in one of the stimuli‐induced ALI models. Interestingly, both similarities and differences in the functions of IFN‐γ were seen when studied using genetic depletion or antibody neutralization and are summarized graphically in Figures S6 and S7.

Measures of injury showed that peak weight loss after LPS was significantly less in the *Ifng* ^−/−^ mice, whereas after *Sp* and PR8, the peak loss and return to baseline were similar between genotypes. The peak weight loss after LPS was also significantly less with IFN‐γ neutralization. In the BAL supernatant, a lower total protein concentration was observed in *Ifng* ^−/−^ mice after *Sp* and PR8 injuries, but not following LPS.

IFN‐γ is required for the changes induced by all three stimuli in myeloid cells. At the peak of LPS‐induced injury (day 4 post‐injury), IFN‐γ is required for full recruitment of neutrophils; however, this difference was only observed in *Ifng* ^−/−^ mice and not following IFN‐γ antibody‐mediated neutralization, which was initiated on day 1 after the neutrophil influx is established (D'Alessio et al., 2009; Zemans & Matthay, 2017). During resolution of all three injuries (day 7, 6, or 15), the *Ifng* ^−/−^ mice have more alveolar macrophages and fewer interstitial and inflammatory macrophages. These changes also occurred following IFN‐γ neutralization after LPS‐induced injury, except for inflammatory macrophages, which are significantly less only at day 4 post LPS, likely due to the timing of antibody delivery. While there are many mechanisms through which these changes in myeloid cells may occur, we postulate that IFN‐γ may enhance injury, which may result in more destruction of alveolar macrophages and greater recruitment of inflammatory monocytes/macrophages from the bone marrow. One mechanism may be similar to that underlying the greater recruitment of inflammatory monocytes and the increased lung injury in juvenile compared to adult mice following infection with influenza A (Coates et al., 2018). These authors provide evidence that a sustained type I IFN response in juvenile mice leads to continued expression of CCL2 (MCP‐1), more inflammatory monocytes entering the lungs, prolonged expression of cytokines, and enhanced and prolonged lung injury in the juvenile mice (Coates et al., 2018). In our studies, the lack of IFN‐γ likely contributes to less CCL2 and less recruitment of inflammatory macrophages, but other effects of IFN‐γ may mediate other aspects of the *Ifng* ^−/−^ mouse phenotype.

In contrast, the contributions IFN‐γ makes to lymphoid cell numbers depends on the stimulus. During resolution of LPS‐induced lung injury, IFN‐γ is also required for a full response of lymphocytes, including CD3^+^, CD4^+^, CD8^+^, and γδ^+^, and NK cells. In *Sp*‐induced injury, *Ifng* ^−/−^ mice have higher numbers of γδ^+^ lymphocytes and fewer NK cells than WT mice. PR8 induces the highest numbers of CD3^+^ lymphocytes during resolution; however, the absence of IFN‐γ does not change the numbers of CD3^+^, CD4^+^, or CD8^+^ lymphocytes. NK T lymphocyte and NK cell numbers are lower in *Ifng* ^−/−^ compared to WT mice after PR8.

Interestingly, antibody neutralization at 1, 2, and 3 days post LPS induced an increase in CD19^+^ B cells at day 4, whereas the *Ifng* ^−/−^ mice had no effect (Figure S7). Since the control antibody appeared to decrease CD19^+^ cells (Figure 1q compared to 4n), an antibody‐mediated nonspecific could contribute to this finding. Lastly, the neutralization experiments did not affect the percentage of effector CD4^+^ or CD8^+^ cells. Collectively, antibody neutralization of IFN‐γ elicits a smaller number of effects compared to the genetic depletion of *Ifng* after LPS‐induced injury. This may be due to the delay in antibody administration until 24 hr after initiation of injury, a partial neutralization of IFN‐γ activity, or IFN‐γ autocrine or local paracrine effects that may not be neutralized by the antibody.

We and others have demonstrated that Tregs play an essential role in the resolution of ALI (D'Alessio et al., 2009; Dial et al., 2017; Lin, Wu, Wang, Xiao, & Xu, 2018; Mock et al., 2014). Following LPS injury, Tregs are increased on day 7 in WT mice (Mock et al., 2019). Despite the fact that Tregs can regulate the expression of IFN‐γ and that IFN‐γ can regulate Treg functions (Overacre‐Delgoffe et al., 2017; Panduro, Benoist, & Mathis, 2018; Sojka & Fowell, 2011), IFN‐γ does not affect the numbers of Tregs after LPS or *S. pneumoniae*. In contrast, IFN‐γ negatively regulates the increase in Tregs induced by influenza. These data suggest that Treg proliferation, recruitment from other sites, or extra‐thymic peripheral expression of Foxp3 during influenza infection may be negatively regulated by IFN‐γ (Olalekan, Cao, Hamel, & Finnegan, 2015). The fact that *Ifng* ^−/−^ mice still retain some ability to increase the numbers of Tregs during influenza, as well as following LPS and Sp injuries, suggests that there are also IFN‐γ‐independent mechanisms for enhancing Treg numbers.

Surprisingly, the total number of epithelial (CD326^+^) and endothelial (CD31^+^) cells were increased in number 7 days post LPS in *Ifng* ^−/−^ mice. One possibility is that IFN‐γ enhances the injury‐ and/or immune‐mediated destruction of epithelial and endothelial cells after LPS injury. Another possibility is that IFN‐γ blunts the pace of endothelial and/or epithelial repair after injury. The data demonstrating earlier or accelerated endothelial proliferation kinetics in *Ifng* ^−/−^ mice after LPS support this hypothesis. In the *Sp*‐induced injury, *Ifng* ^−/−^ mice demonstrated increased numbers of endothelial cells during resolution, whereas after PR8‐induced injury, the *Ifng* ^−/−^ mice had higher numbers of epithelial cells compared to WT controls. Importantly, the differences in isolation conditions or in endothelial or epithelial cell viability between genotypes may complicate the differences measured in these cell types and should be considered.

Overall, IFN‐γ enhances injury and may delay recovery, as measured by return to pre‐injury weight, BAL protein levels, and clearance of inflammatory cells. The mechanisms by which IFN‐γ coordinates its impact upon ALI resolution are likely multifactorial. First, IFN‐γ may act by directly signaling immune cell populations, through the production of downstream chemokines, cytokines, and their mediators, or by inducing apoptosis (Schroder et al., 2004). IFN‐γ may target multiple cell types, including alveolar macrophages. Second, in contrast to the myeloid cell kinetics, which are similar for the three different stimuli, the differences in lymphoid responses between ALI stimuli likely underline injury‐specific requirements for the recruitment of lymphocytes. However, we cannot exclude the possibility that the differences in the roles of IFN‐γ between injuries are due to the time points examined, which may not represent an identical point in the resolution process.

Comparing and contrasting the results of genetic deletion or antibody neutralization suggests that temporal regulation of IFN‐γ during injury is a crucial factor in IFN‐γ’s functions. The lack of IFN‐γ activity early during injury (within the first 24 hr) is essential in impacting neutrophil recruitment at Day 4. Administering antibody neutralization at days 1, 2, and 3 does not affect neutrophil numbers at Day 4, but exerts an effect similar to genetic deletion on alveolar macrophages and inflammatory macrophages.

Our data support and expand the work of others who have suggested that dampening the function of IFN‐γ may be beneficial in ALI. One prospective observational cohort study performed a cluster analysis of 20 potential biomarkers in plasma of ARDS patients and found different ARDS phenotypes (Bos et al., 2017). This study identified a set of four biomarkers, Angiopoietin 1/2, IFN‐γ, IL‐6, and plasminogen activator inhibitor 1, which could categorize two phenotypic groups of ARDS patients. The “reactive” phenotype group, which had higher levels of these four mediators, including IFN‐γ, had significantly higher mortality than the “uninflamed” phenotype (Bos et al., 2017). The authors of this study suggested that this “reactive” ARDS phenotype may benefit from targeted pharmacological interventions (Bos et al., 2017). Other work also supports CD8^+^ cells as contributing to IFN‐γ production and lung injury (Ramana et al., 2015; Wlodarczyk et al., 2013). These studies, along with ours, demonstrate that potential immunotherapy to block or neutralize IFN‐γ could be useful in controlling severe lung inflammation. Recently, in the United States, a recombinant IFN‐γ blocking antibody (Emapalumab) was approved for patients with primary hemophagocytic lymphohistiocytosis (Al‐Salama, 2019).

In summary, our results illustrate the complexity of lung injury and its resolution and provide insight into the effects of one cytokine on lung injury and resolution. Uncoupling IFN‐γ’s effect on peak injury from its effect on resolution remains an incomplete task. Understanding the interactions between inflammation and repair is critical to identify approaches that minimize tissue damage without adversely altering the host response.

## CONFLICT OF INTEREST

The authors have declared that no conflict of interest exists.

## AUTHORS’ CONTRIBUTIONS

JRM, MKT, CFD, and RSH: conceived and designed experiments. JRM, MKT, CFD, JTC, and RSH performed experiments and analysis. JRM and CMD wrote the manuscript and provided creative input. All authors have read and approved the final manuscript.
