# Supplementary material for: Effects of IFN‐γ on immune cell kinetics during the resolution of acute lung injury
Source: Physiol Rep. 2020 Feb 15;8(3):e14368. doi: 10.14814/phy2.14368 (PMC7023890; doi:10.14814/phy2.14368)
Supplement: Supplementary file 1 [file PHY2-8-e14368-s001.docx]

**SUPPLEMENTAL DATA**

Supplemental Figure 1


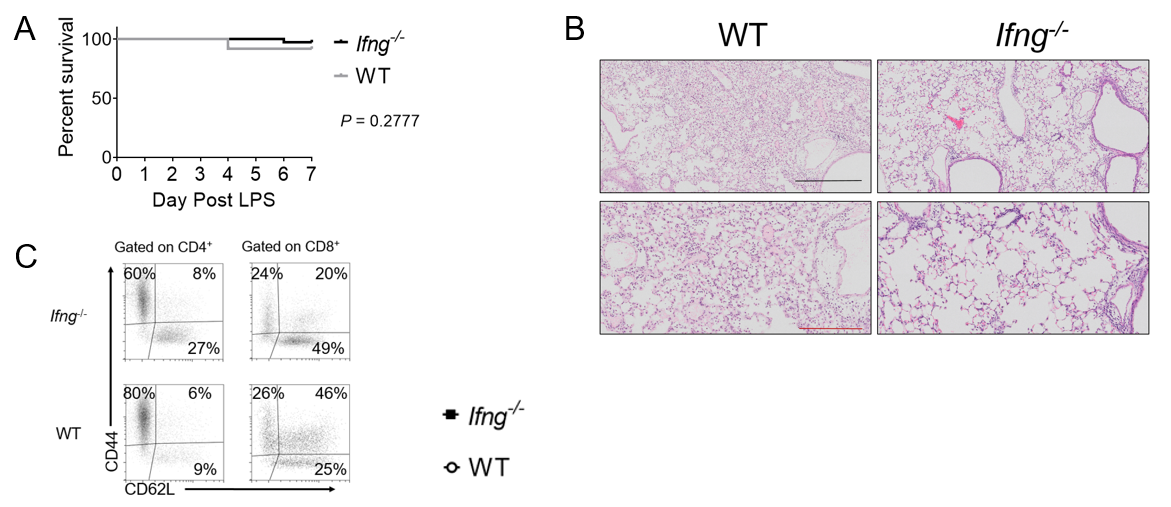


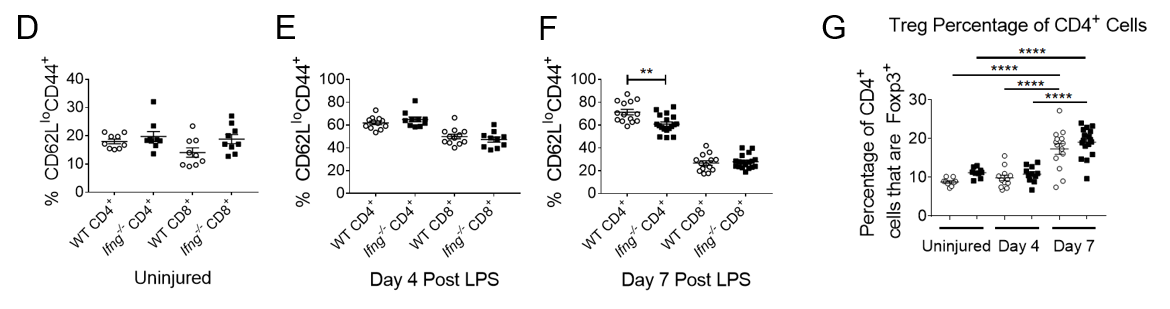


**
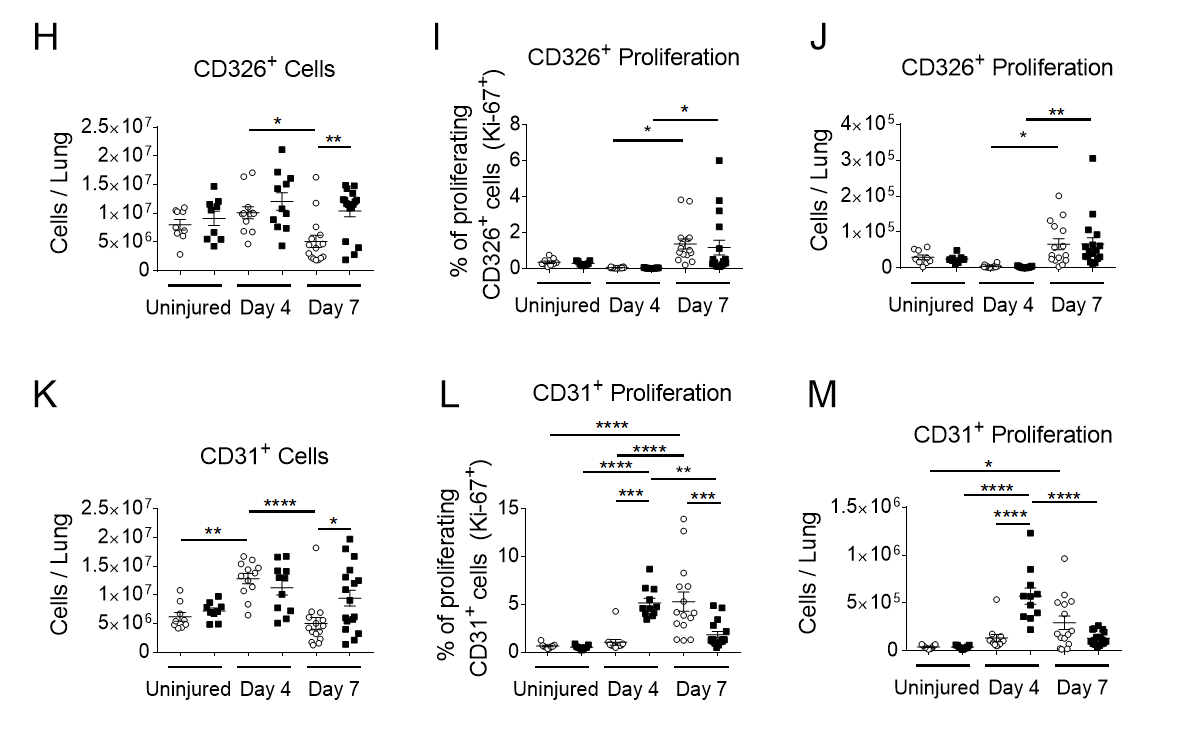
**

**Supplemental Figure 1.** ***Ifng^-/-^* mice have a lower percentage of effector CD4^+^ cells and an earlier increase in CD31^+^ cell percent proliferation during resolution of LPS-induced ALI.** (A) Survival of *Ifng^-/-^* mice and WT mice following LPS administration (n = 39 *Ifng^-/-^* mice; n = 37 for WT mice, combining 4 independent experiments). *P* value by Log-rank (Mantel-Cox) test. (B) Representative H&E lung sections demonstrate increased cellularity 4 days after LPS-induced lung injury in WT mice. Black bar 500 μM and maroon bar 250 μM. (C) Representative flow cytometric dot plot and gating of CD4^+^ and CD8^+^ lymphocytes in the lung gating for CD44 and CD62L expression. Plots are representative of at least 2 independent experiments. (D-F) Percentage of CD4^+^ and CD8^+^ cells in the lung that express high levels of CD44 and low levels of CD62L at uninjured (D), day 4 (E) or day 7 (F) post LPS. (G) Percentage of CD4^+^ cells that express Foxp3^+^ at day 4 or 7 post LPS (n = 9-17 per genotype) (H-M) Total epithelial (CD326^+^) and endothelial (CD31^+^) cells and their percentage of proliferation (Ki-67^+^) and the number of proliferating cells in single-cell lung suspensions determined using a published flow cytometric approach ([39](#_ENREF_39)); (n = 10-18 per group, combined from at least 2 independent experiments). Data are expressed as the mean ± SEM. *P* values determined by one-way or two-way ANOVA with the Holm-Sidak multiple comparison test. * *P* < 0.05, ** *P* < 0.01 *** *P* < 0.001, **** *P* < 0.0001.

Supplemental Figure 2


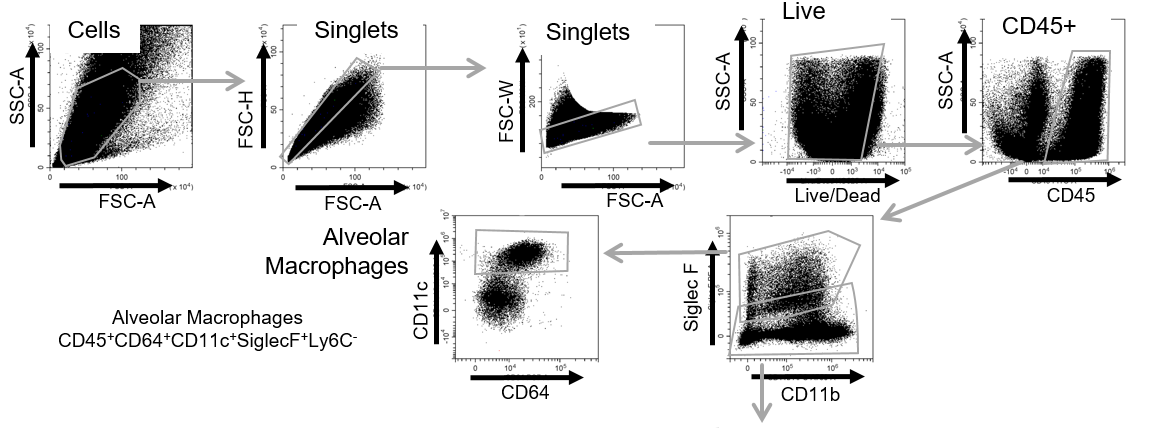

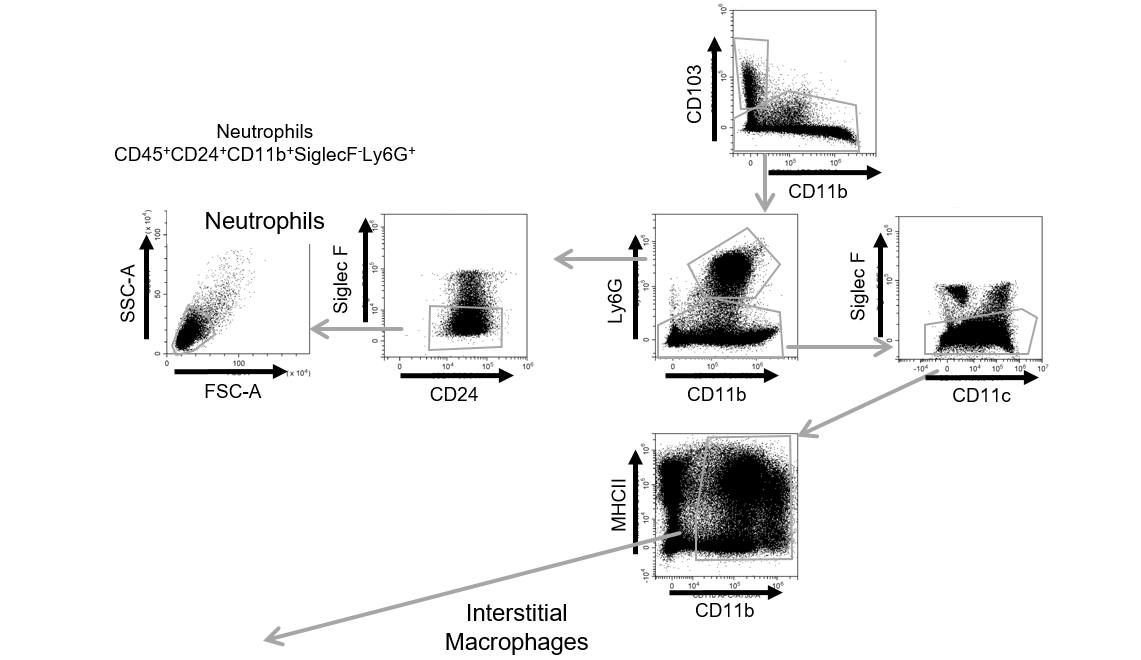

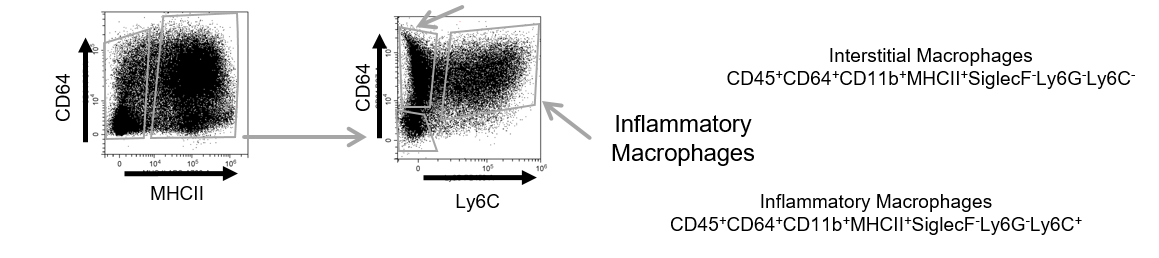


**Supplemental Figure 2. Flow cytometric gating method for the identification of myeloid cells.** The flow cytometric dot plots and gating scheme used for identification of myeloid cell populations adapted from a previous report ([31](#_ENREF_31)). Gating and dot plot results are representative of three independent experiments.

Supplemental Figure 3


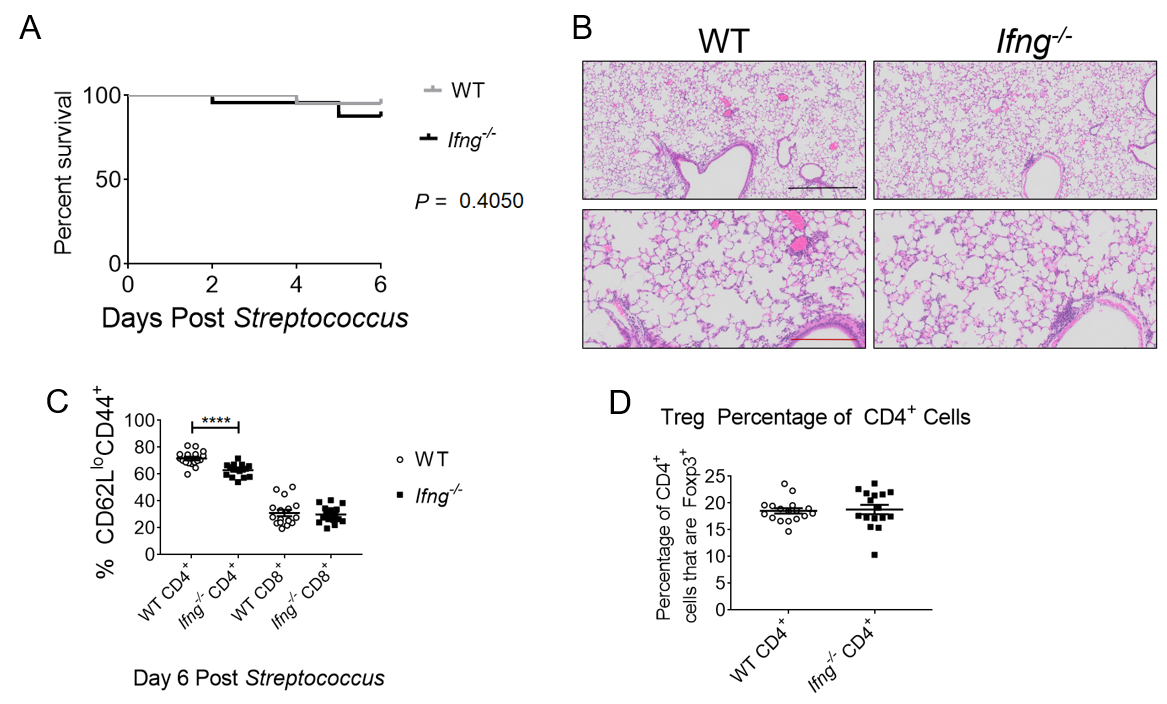

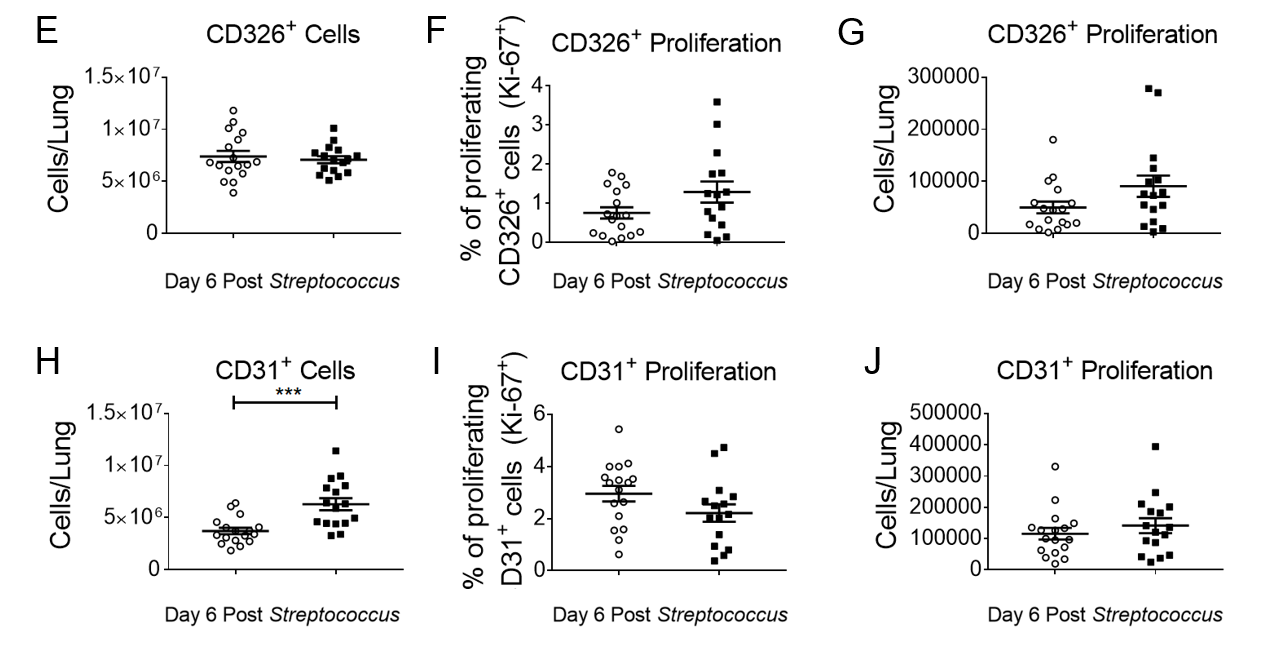


**Supplemental Figure 3.** ***Ifng^-/-^* mice have a lower percentage of effector CD4^+^ cells and a larger number of CD31^+^ cells during resolution of *Streptococcus pneumoniae*-induced ALI.** (A) Survival of *Ifng^-/-^* mice and WT mice following *Streptococcus pneumoniae* (*Sp*) administration (n = 21 for *Ifng^-/-^* mice; n = 19 for WT mice, combining 2 independent experiments). *P* value by Log-rank (Mantel-Cox) test. (B) Representative H&E lung sections demonstrate similar cellularity 6 days after *Sp*-induced lung injury in WT mice. Black bar 500 μM and maroon bar 250 μM. (C) Percentage of CD4^+^ and CD8^+^ cells in the lung that express high levels of CD44 and low levels of CD62L at 6 days post-*Sp* (n = 16-17 per genotype, data combined from 2 independent experiments). Percentages of effector CD4^+^ and CD8^+^ lymphocytes as determined by CD44^+^ CD62L^lo^ surface markers. (D) Percentage of CD4^+^ cells that express Foxp3^+^ at day 6 post *Sp* (n = 16-17 per genotype). (E-J) Total epithelial (CD326^+^) and endothelial (CD31^+^) cells and their percentage of proliferation (Ki-67^+^) and the number of proliferating cells in single-cell suspensions determined using a published flow cytometric approach ([39](#_ENREF_39)); (n = 15-16 per group, combined from at least 2 independent experiments). Data are expressed as the mean ± SEM. *P* values determined by either Mann Whitney rank sum test or one-way ANOVA with the Holm-Sidak, multiple comparison tests. *** *P* < 0.001, **** *P* < 0.0001.

Supplemental Figure 4


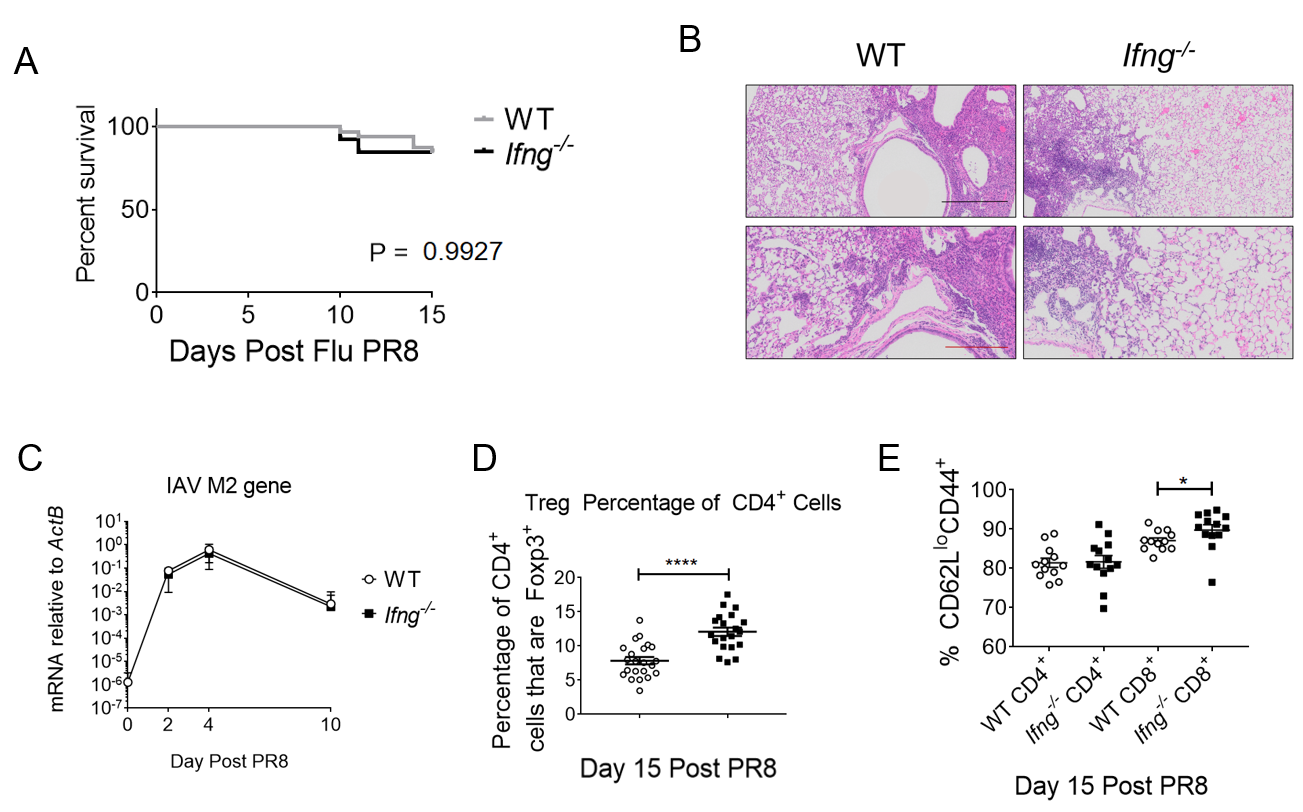

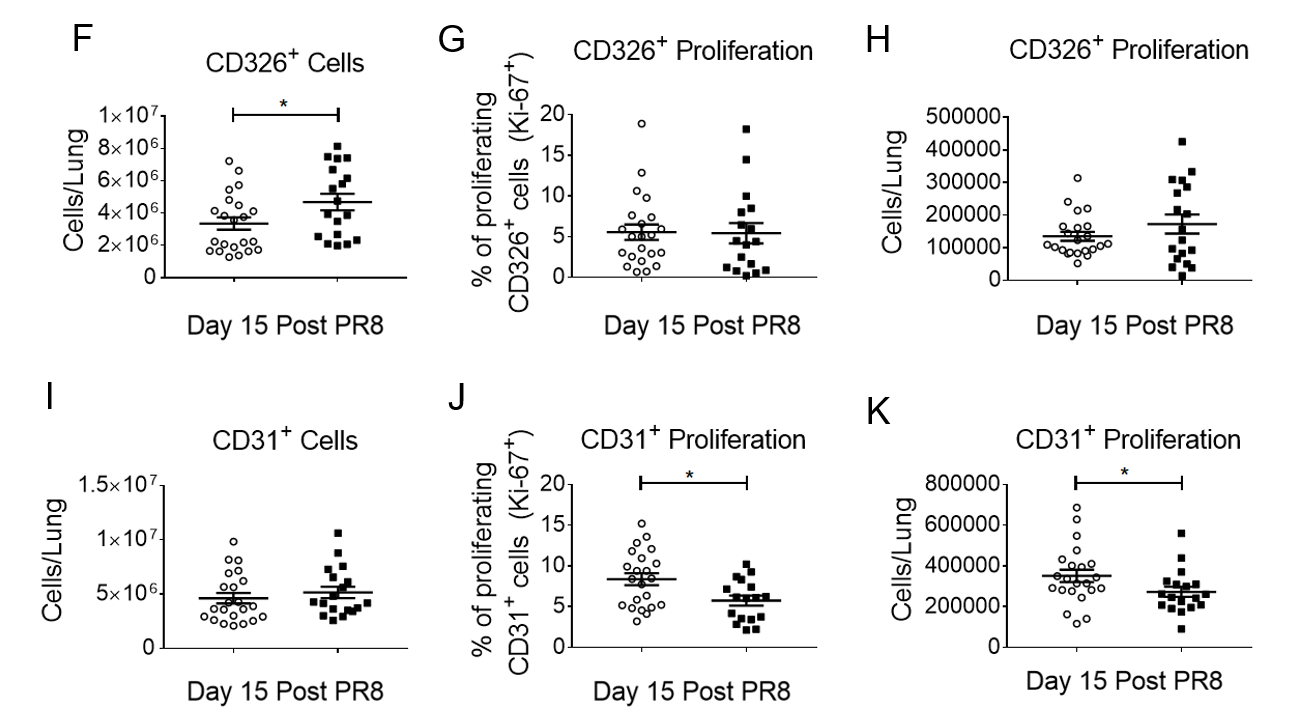


**Supplemental Figure 4.** ***Ifng^-/-^* mice have more CD326^+^ cells during a time point of resolution from Influenza A H1N1-induced ALI.** (A) Survival of *Ifng^-/-^* mice and WT mice following Influenza A H1N1 PR8 (PR8) administration (n = 26 for *Ifng^-/-^* mice; n = 31 for WT mice, combining 3 independent experiments). *P* value by Log-rank (Mantel-Cox) test. (B) Representative H&E lung sections demonstrate similar cellularity 15 days after PR8-induced lung injury between genotypes. Black bar 500 μM and maroon bar 250 μM. (C) Viral infection level determination between genotypes measured by RT-qPCR of influenza A M2 gene from lung homogenates at day 2, 4 or 10 post PR8 (n = 6-8 per time point and genotype, combined data from two independent experiments). (D) Percentage of CD4^+^ cells that express Foxp3^+^ at day 15 post PR8 (n = 12-14 per genotype). (E) Percentage of CD4^+^ and CD8^+^ cells in the lung that express high levels of CD44 and low levels of CD62L at 15 days post PR8 (n = 12-13 per genotype and combined from 2 independent experiments). (F-K) Total epithelial (CD326^+^) and endothelial (CD31^+^) cells and their percentage of proliferation (Ki-67^+^) and the number of proliferating cells in single-cell suspensions determined using a published flow cytometric approach ([39](#_ENREF_39)). (n = 15-16 per group, combined from at least 2 independent experiments). Data are expressed as the mean ± SEM. *P* values determined by either Mann Whitney rank sum test or one-way ANOVA with the Holm-Sidak, multiple comparison tests. * *P* < 0.05, **** *P* < 0.0001.

Supplemental Figure 5


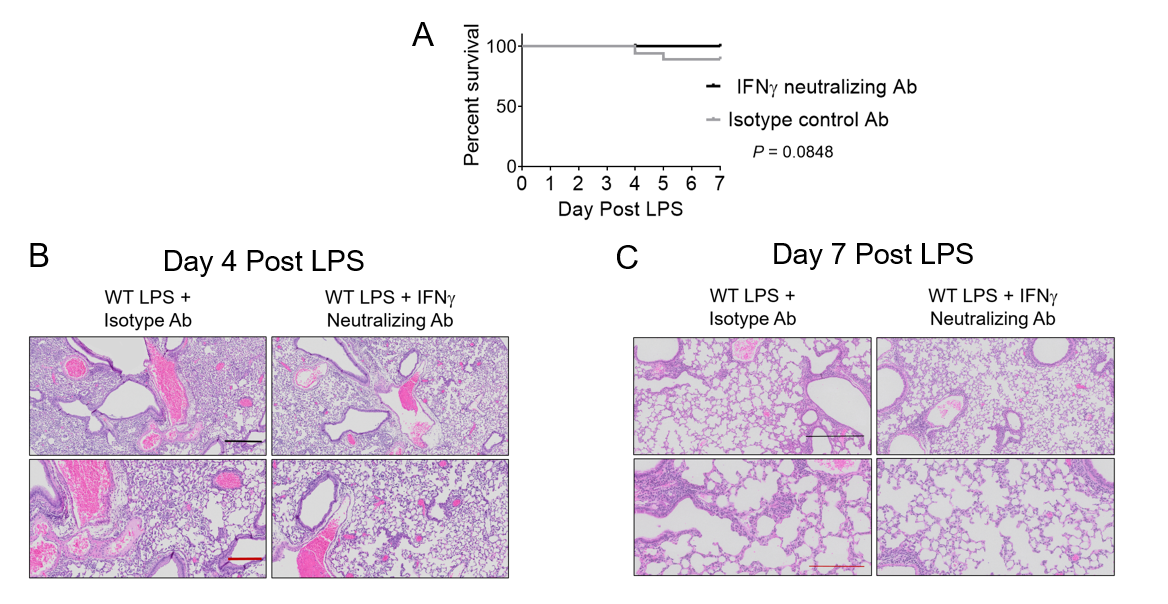


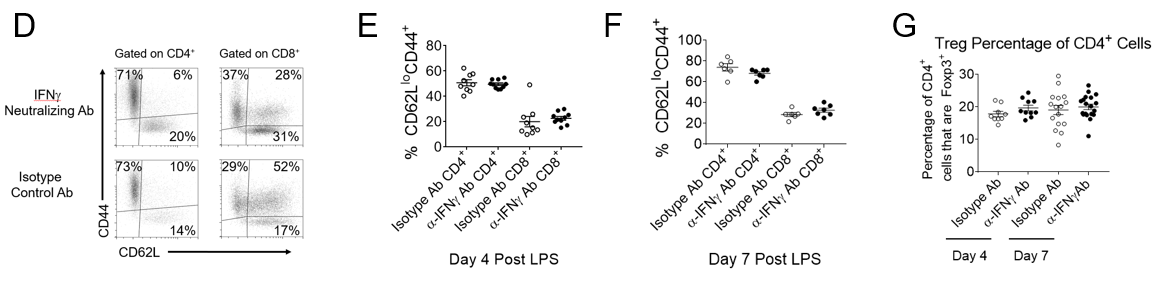

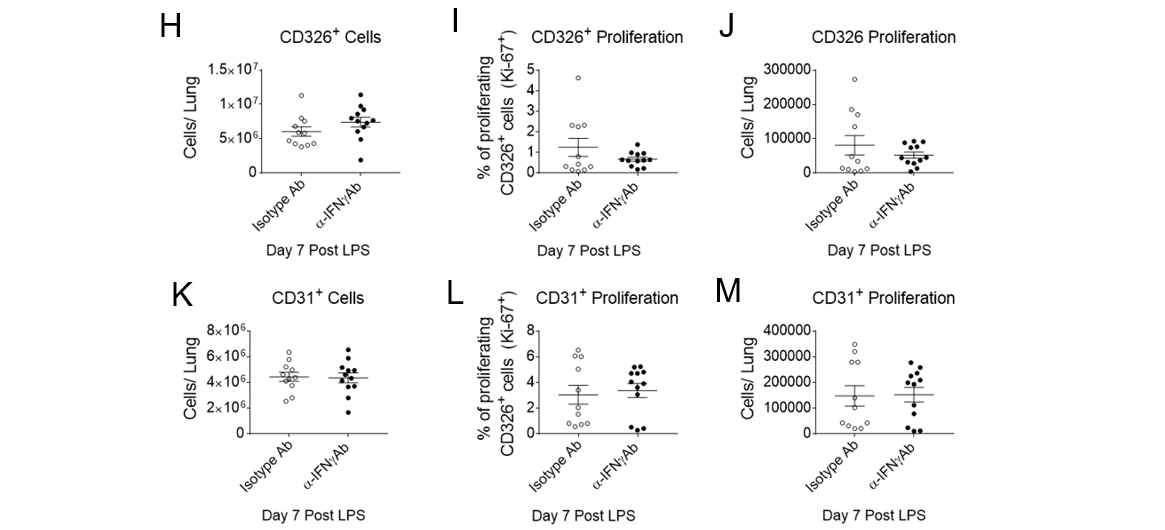


**Supplemental Figure 5.** **Effect of** **IFN-**γ **antibody neutralization on immune subset numbers and epithelial proliferation during resolution of LPS-induced ALI.** (A) Survival of WT mice administered an IFN-γ neutralizing antibody or an isotype control antibody following LPS administration (n = 32 for IFN-γ neutralizing antibody; n = 33 for isotype control antibody, combining 2 independent experiments). *P* value by Log-rank (Mantel-Cox) test. (B-C) Representative H&E lung sections demonstrate similar cellularity 4 (B) and 7 (C) days after LPS-induced lung injury in both conditions. Black bar 500 μM and maroon bar 250 μM. (D) Representative flow cytometric dot plot and gating of CD4^+^ and CD8^+^ cells in the lung for CD44 and CD62L expression. (E-F) Percentages of CD4^+^ and CD8^+^ cells in the lung that express high levels of CD44 and low levels of CD62L at 4 (E) or 7 (F) days post LPS (n = 6-7 per group, representative of 2 independent experiments). (G) Percentage of CD4^+^ cells that express Foxp3^+^ on day 4 or 7 post LPS. (H-M) Total epithelial (CD326^+^) and endothelial (CD31^+^) cells and their percentage of proliferation (Ki-67^+^) and the number of proliferating cells in single cell suspensions determined using a published flow cytometric approach ([39](#_ENREF_39)). (n = 12 per group, combined from at least 2 independent experiments). Data are expressed as the mean ± SEM. *P* values determined by either Mann Whitney rank sum test, one-way or two-way ANOVA with the Holm-Sidak, multiple comparison tests.

Supplemental Figure 6


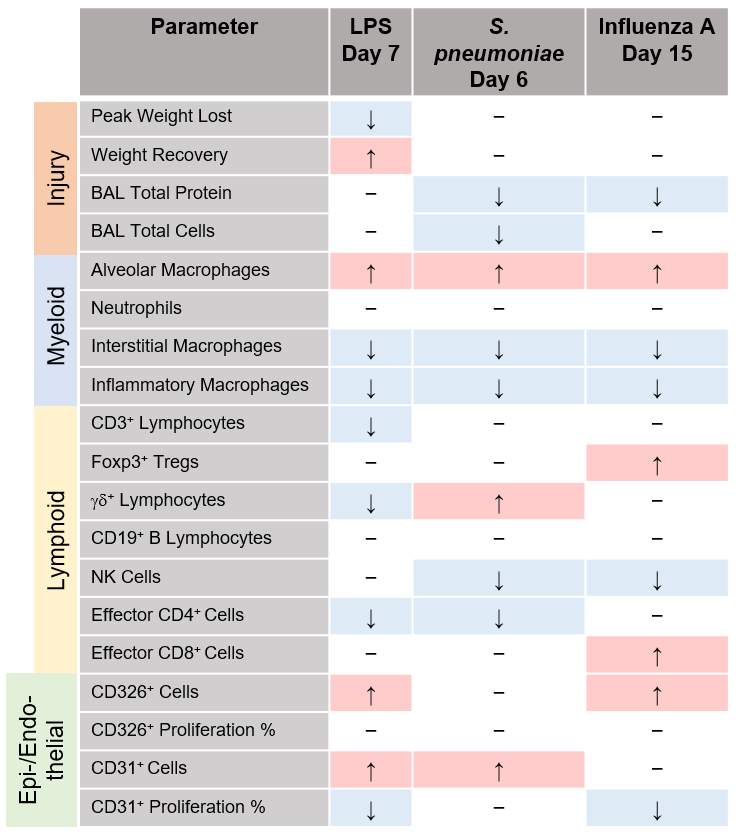


**Supplemental Figure 6.** **Differences in the inflammatory and immune cell responses between wild-type and *Ifng^-/-^* mice during resolution of acute lung injury.** Wild-type and *Ifng^-/-^* mice were subjected to either LPS, *Sp*, or PR8 acute lung injury. *Ifng^-/-^* mice results compared to wild-type mice are shown for all three injury models for injury parameters, immune, epithelial, or endothelial cell population numbers. Data obtained from Figures 1-3 and Supplemental Figures 1, 3, and 4. A ↑ with a red box signifies an increase in *Ifng^-/-^* mice compared to WT mice, while a ↓ with a blue box signifies a decrease in *Ifng^-/-^* mice compared to WT mice.

Supplemental Figure 7

**
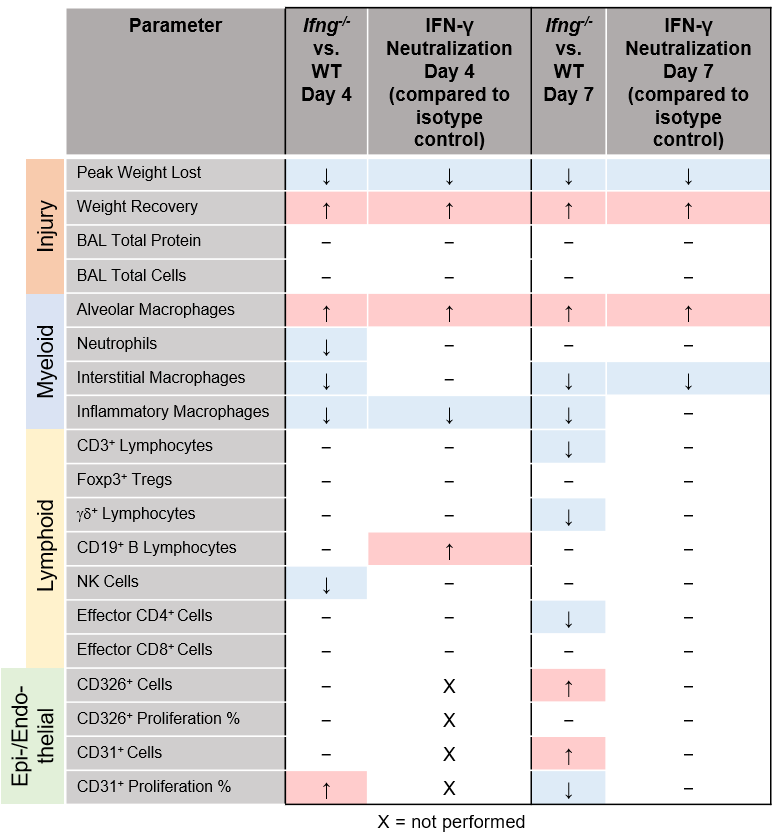
**

**Supplemental Figure 7.** **IFN-**γ **knockout and IFN-**γ **antibody neutralization effects on inflammatory and immune cell responses after LPS ALI.** Wild-type and *Ifng^-/-^* mice were subjected to either LPS acute lung injury. *Ifng^-/-^* mice results compared to wild-type mice are shown for injury parameters, immune, epithelial, or endothelial cell population numbers on days 4 and 7 (columns 2 and 4; Figure 1 and Supplemental Figure 1). WT mice were administered (intraperitoneally) an IFN-γ neutralizing antibody or isotype control after LPS-induced ALI at days 1, 2, and 3 post LPS–induced injury. Changes in injury parameters, immune, epithelial or endothelial cell population numbers at days 4 or 7 post LPS-induced ALI for the IFN-γ neutralizing antibody group to mice administered isotype control are shown in columns 3 and 5 (Figure 4 and Supplemental Figure 5). A ↑ with a red box signifies an increase in *Ifng^-/-^* mice, or WT mice administered an IFN- γ neutralizing antibody compared to WT mice or WT mice administered an isotype control antibody. A ↓ with a blue box signifies a decrease in *Ifng^-/-^* mice, or WT mice administered an IFN-γ neutralizing antibody compared to WT mice or WT mice administered an isotype control antibody.

Supplemental Table 1

| **Antibody** | **Clone** | **Company** | **Catalog #** |
| --- | --- | --- | --- |
| ST2 PE | D1H9 | BioLegend | 145304 |
| CD103 PE/Cy7 | 2E7 | BioLegend | 121426 |
| CD4 Alexa 700 | GK1.5 | BioLegend | 100429 |
| Foxp3 Alexa 647 | 150D | BioLegend | 320014 |
| CD45 FITC | 30-F11 | BioLegend | 103108 |
| Siglec F PE | E50-2440 | BD Biosciences | 552126 |
| CD24 PerCP-Cy5.5 | M1/69 | BioLegend | 101824 |
| CD64 PE-Cy7 | X54-5/7.1 | BioLegend | 139314 |
| Ly6G APC | 1A8 | BioLegend | 127614 |
| MHCII Alexa 700 | M5/114.15.2 | BioLegend | 107618 |
| CD11b APC Cy7 | M1/70 | BioLegend | 101216 |
| Ly6C PB | HK1.4 | BioLegend | 128024 |
| Zombie aqua | - | BioLegend | 423102 |
| CD11c BV 605 | N418 | BioLegend | 117334 |
| CD103 BV786 | M290 | BD Biosciences | 564322 |
| CD104 FITC | 346-11A | BioLegend | 123606 |
| Ki67 PE | B56 | BD Biosciences | 556027 |
| CD326 PE-Dazzle | G8.8 | BioLegend | 118236 |
| CD14 PE-Cy7 | Sa14-2 | BioLegend | 123316 |
| Zombie NIR | - | BioLegend | 423106 |
| CD31 PB (BV421) | 390 | BioLegend | 102422 |
| T1 alpha biotin | 8.1.1 | BioLegend | 127404 |
| Streptavidin BV605 | - | BioLegend | 405229 |
| CD45 BV785 | 30-F11 | Biolegend | 103149 |
| CD104 APC | 346-11A | Biolegend | 123612 |
| FcγRIII/FcγRII | 2.4G2 | BD Biosciences | 553141 |
| γ/δ PerCP-Cy5.5 | GL3 | BioLegend | 118108 |
| CD3 FITC | 145-2C11 | BioLegend | 100306 |
| CD8a PE-CF594 | 53.6.7 | BioLegend | 100762 |
| NK-1.1 BV785 | PK136 | BioLegend | 108749 |
| LEAF anti-mouse IFN-γ | AN-18 | BioLegend | 517904 |
| LEAF Rat IgG1 κ isotype | RTK2017 | BioLegend | 400414 |

**Supplemental Table 1.** Fluorescent-conjugated antibodies and neutralization antibodies used in this study. Low endotoxin azide free = LEAF.
